# Estimating the disease burden of methicillin-resistant *Staphylococcus aureus* in Japan: Retrospective database study of Japanese hospitals

**DOI:** 10.1371/journal.pone.0179767

**Published:** 2017-06-27

**Authors:** Hironori Uematsu, Kazuto Yamashita, Susumu Kunisawa, Kiyohide Fushimi, Yuichi Imanaka

**Affiliations:** 1 Department of Healthcare Economics and Quality Management, Graduate School of Medicine, Kyoto University, Yoshida Konoe-cho, Sakyo-ku, Kyoto City, Kyoto, Japan; 2 Department of Health Policy and Informatics, Graduate School of Medicine, Tokyo Medical and Dental University, Yushima, Bunkyo-ku, Tokyo, Japan; Wadsworth Center, UNITED STATES

## Abstract

**Objectives:**

The nationwide impact of antimicrobial-resistant infections on healthcare facilities throughout Japan has yet to be examined. This study aimed to estimate the disease burden of methicillin-resistant *Staphylococcus aureus* (MRSA) infections in Japanese hospitals.

**Design:**

Retrospective analysis of inpatients comparing outcomes between subjects with and without MRSA infection.

**Data source:**

A nationwide administrative claims database.

**Setting:**

1133 acute care hospitals throughout Japan.

**Participants:**

All surgical and non-surgical inpatients who were discharged between April 1, 2014 and March 31, 2015.

**Main outcome measures:**

Disease burden was assessed using hospitalization costs, length of stay, and in-hospital mortality. Using a unique method of infection identification, we categorized patients into an anti-MRSA drug group and a control group based on anti-MRSA drug utilization. To estimate the burden of MRSA infections, we calculated the differences in outcome measures between these two groups. The estimates were extrapolated to all 1584 acute care hospitals in Japan that have adopted a prospective payment system.

**Results:**

We categorized 93 838 patients into the anti-MRSA drug group and 2 181 827 patients into the control group. The mean hospitalization costs, length of stay, and in-hospital mortality of the anti-MRSA drug group were US$33 548, 75.7 days, and 22.9%, respectively; these values were 3.43, 2.95, and 3.66 times that of the control group, respectively. When extrapolated to the 1584 hospitals, the total incremental burden of MRSA was estimated to be US$2 billion (3.41% of total hospitalization costs), 4.34 million days (3.02% of total length of stay), and 14.3 thousand deaths (3.62% of total mortality).

**Conclusions:**

This study quantified the approximate disease burden of MRSA infections in Japan. These findings can inform policymakers on the burden of antimicrobial-resistant infections and support the application of infection prevention programs.

## Introduction

Antimicrobial-resistant (AMR) infections present a growing threat to global health [1], and are predicted to be the leading cause of death in the near future [2]. These infections not only severely compromise human health, but also place a strain on healthcare systems and require an excessive consumption of resources [3].

The relative proportion of AMR infections varies among regions [1], and the majority of AMR infections in Japan are due to methicillin-resistant *Staphylococcus aureus* (MRSA) [4]. An analysis of 806 hospitals conducted as part of the Japan Nosocomial Infections Surveillance program in 2015 attributed 93.6% of newly detected AMR infections to MRSA, followed by penicillin-resistant *Streptococcus pneumoniae* (3.64%) and carbapenem-resistant *Enterobacteriaceae* (1.75%) [5]. MRSA infections may therefore be considered the representative disease of AMR infections in Japan.

Although policymakers require accurate information on the prevalence and total disease burden of AMR infections in order to develop and implement countermeasures, there are difficulties in providing this information. Several studies have estimated the disease burden of these infections in other countries [6–8], but the generalizability of their findings is problematic due to their reliance on data from a small sample of hospitals. However, developing new surveillance schemes to collect information on disease burden from a large number of hospitals requires a vast amount of time and resources. It would therefore be beneficial if an existing system can be repurposed to enable estimates of disease burden through rapid and low-cost analysis.

A nationwide administrative claims database may facilitate the quantification of AMR infection burden with minimal effort. Utilizing a large-scale secondary database offers several advantages to researchers, such as waiving the need to develop a surveillance system and supplying large sample sizes to strengthen the external validity of findings [9]. However, researchers often face problems in utilizing such a database for assessing AMR infections due to a lack of clinical data, such microbiological information.

We have previously developed and validated a method for identifying hospital-acquired infections based on antibiotic utilization patterns from administrative claims data [10]. By applying a similar technique, it may be possible to estimate the general disease burden of AMR infections in the majority of Japanese hospitals by using a national administrative claims database.

The aims of this study were to quantify the clinical and economic burden of MRSA infections in inpatients using a Japanese national administrative claims database comprising data from 1133 hospitals, and to estimate the total current disease burden in acute care hospitals throughout Japan.

## Materials and methods

### Data source

Data were obtained from a Diagnosis Procedure Combination (DPC) database containing administrative claims data that are periodically collected from voluntarily participating hospitals [11]. The DPC system is a patient case-mix system used in the determination of reimbursements for acute care hospitals, and these data encompass approximately half of all inpatient admissions to acute care hospitals in Japan. The participating hospitals submit electronic data to the DPC Research Group (which is funded by Japan’s Ministry of Health, Labour and Welfare) to be applied in disease management analyses [12]. DPC data consist of summarized clinical information and detailed healthcare claims data. The clinical information includes patient age, sex, major diagnoses, admission and discharge dates, and outcomes at discharge. Diseases are classified using International Classification of Diseases, 10th Revision (ICD-10) codes. The database also includes information on the detailed processes of care, such as diagnostic tests, drug administration, and all surgical and interventional procedures with their specific dates of implementation and corresponding costs.

### Study inclusion and exclusion criteria

We first selected all inpatients who had been discharged between April 1, 2014 and March 31, 2015 for inclusion in the study. We excluded patients if they had missing values in sex, age, death, admission and discharge dates, and cost data.

### Identification of MRSA infection cases

The DPC database does not include microscopy or laboratory-based information on the etiologic agents of infections, thereby precluding the direct identification of MRSA infections. As an alternative method of identification, we regarded patients who had been administered anti-MRSA drugs as being infected with MRSA according to the criteria described below. The guidelines issued by the Japanese Association for Infectious Diseases and the Japanese Society of Chemotherapy recommend five anti-MRSA drugs (vancomycin, teicoplanin, daptomycin, linezolid, and arbekacin) for the treatment of MRSA infections [4,13]. In addition, they recommend first-generation cephalosporins (cefazolin and cephalexin) as first-line therapy for treating methicillin-sensitive S. aureus infections, for which the use of anti-MRSA drugs is not covered under Japanese health insurance [13].

To account for the possibility of non-MRSA infected patients being administered anti-MRSA drugs as empirical therapy, we only considered patients who were administered these drugs for four days or more as MRSA infection cases, as described in our previous study [4].

### Cost estimation

Hospitalization costs were calculated from the payer’s perspective. The hospitals in the DPC database have adopted a per diem prospective payment system (PDPS) where reimbursements are pre-determined according to patient case-mix classifications that involve a combination of both diagnoses and procedures. Although hospitals are generally reimbursed for treating the majority of inpatients under the DPC-PDPS, they can also be reimbursed under a fee-for-service system for patients admitted to intensive care units, who had undergone unconventional surgeries, or had overly long hospitalization durations [14]. In this study, we generally estimated hospitalization costs using PDPS fees. In cases that required reimbursements under the fee-for-service system, we calculated the summary of all fees for medical services provided during hospitalization using a previously described method [15, 16]. Hospitalization costs included basic and specialized inpatient care, initial consultation and examination, imaging services, pharmacy, injections, treatments, invasive procedures, and pre-discharge consultation. The fees were summarized in Japanese yen and converted to US dollars (US$1 = 105) using the mean purchasing power parity in 2014 [17].

### Disease burden

As indicators of the clinical and economic burden of MRSA infections, the primary outcome measures were hospitalization costs, length of stay (LOS) and in-hospital (all-cause) mortality. The study sample was first divided into an anti-MRSA drug group, a control group, and a non-infection group. Patients were included in the anti-MRSA drug group if they had been administered any of the five stipulated anti-MRSA drugs for four days or more. Patients were included in the control drug group if they had been administered any antibiotics except the anti-MRSA drugs for four days or more. All other patients were included in the non-infection group.

To estimate the disease burden in the anti-MRSA drug group relative to the control group, we calculated the mean ratio of each outcome by dividing the mean outcome values in the anti-MRSA drug group by the mean outcome values in the control group. To estimate the total disease burden of MRSA infections, we first calculated the differences in mean outcome values between the anti-MRSA drug group and the control group; the results were then multiplied by the number of MRSA infection cases in each of the three-digit ICD-10 codes of major diagnoses for surgical and non-surgical inpatients, and summarized using the following formula:

$$\text{Burden estimation = }\sum_{i}\left\{\left(\text{Mean outcome MRSA}i–\text{ Mean outcome Control}i\right)\text{ * Case MRSA}i\;\right\},$$

where *i* refers to the three-digit ICD-10 codes for surgical and non-surgical inpatients.

We calculated the total disease burden in two populations: the first population (Population A) was obtained from the DPC database used in this study, and the second population (Population B) comprised inpatients admitted to all hospitals in Japan that have adopted the DPC/PDPS; data for the latter were obtained from a public survey conducted by a commercial organization for investigating medical service fees [18]. As a reference, we generated a version of Population A that was adjusted for age and sex. This was calculated by summarizing the burden of MRSA infections according to each three-digit ICD-10 codes, and adjusting for three age categories (≤64y, 65–74 y, and ≥75 y) and sex. In addition, we performed a differential analysis for surgical and non-surgical inpatients due to the potential difference in resource allocation [19].

### Inter-hospital variations in MRSA infections

To investigate the differences in estimated MRSA incidence among hospitals, we also calculated observed-to-expected (O/E) ratios [20]. First, we calculated the standard MRSA incidence by dividing all cases in the anti-MRSA drug group by all inpatients (including the non-infection cases) in the study population. Second, we multiplied the standard MRSA incidence by the number of cases in each ICD-10 codes (surgical and non-surgical inpatients) to calculate the expected number (“E”) of MRSA cases in each hospital. Third, we set the number of cases in the anti-MRSA drug group in each hospital as the number of observed cases (“O”). Finally, we calculated the O/E ratios of each hospital using the following formula:

$$\text{O/E ratio = }\frac{\text{Number of cases in the anti-MRSA drug group}}{\sum_{\text{i}}\text{(Standard MRSA incidence ​i* Number of all cases i)}},$$

where *i* refers to the three-digit ICD-10 codes for surgical and non-surgical inpatients.

All statistical analyses were performed using R statistical software (version 3.2.1). The Ethics Committee of Kyoto University Graduate School of Medicine approved the collection and analysis of DPC data (Approval Number: R0135). In accordance with the Japanese Ethical Guidelines for Medical and Health Research involving Human Subjects, our study waived the need for informed consent.

## Results

We identified a total of 7 794 604 candidate subjects using the inclusion criteria. We then excluded 22 554 (0.29% of all patients) patients with missing values in sex (n = 1), age (n = 32), and cost data (n = 22 522). After excluding these patients, the study sample consisted of 7 772 050 inpatients admitted to 1133 hospitals. Among these, 93 838 patients (1.21% of all eligible patients) were categorized into the anti-MRSA drug group and 2 181 827 patients were categorized into the control group; the remaining 5 496 385 patients were categorized into the non-infection group.

### Demographic characteristics

Table 1 summarizes the demographic characteristics and outcomes of the study sample. The mean ages of patients in the anti-MRSA drug group, control group, and non-infection group were 66.8 years, 62.6 years, and 59.3 years, respectively. The mean and median hospitalization costs in the anti-MRSA drug group were US$33 548 and US$21 399, respectively, and were higher than those of the control and non-infection groups. Similarly, the mean and median LOS in the anti-MRSA drug group were 75.7 days and 51 days, respectively, and were also higher than those of the control and non-infection groups. In-hospital mortality in the anti-MRSA drug group was also higher than in the other groups at 22.9%. The mean difference (incremental outcome) in mean hospitalization costs, LOS, and mortality between the anti-MRSA drug group and the control group were US$23 569, 50.0 days, and 16.6%, respectively. The mean ratios of the hospitalization costs, LOS, and mortality in the anti-MRSA drug group to the control group were 3.43, 2.95, and 3.66, respectively.

**Table 1:** Demographic characteristics of 7 772 050 inpatients.

	All	Anti-MRSA drug group^a^	Control group^b^	Non-infection group^c^	Mean difference^d^	Mean ratio^e^
Number of hospitals, n	1133	1119	1132	1133	-	-
Number of patients, n (%)	7 772 050 (100)	93 838 (1.21)	2 181 827 (28.1)	5 496 385 (70.7)	-	-
Mean age, years (SD)	60.3 (24.4)	66.8 (21.0)	62.6 (25.1)	59.3 (24.0)	-	-
Female sex, n (%)	3 663 760 (47.1)	35 579 (37.9)	1 012 860 (46.4)	2 615 321(47.6)	-	-
Mean hospitalization costs, US$ (SD)	6362 (10 210)	33 548 (46 537)	9979 (12 760)	4626 (5359)	23 569	3.43
Median hospitalization costs, US$ (IQR)	3914 (1954–7211)	21 399 (11 435–39 437)	6143 (3468–11 735)	3160 (1594–5483)	-	-
Mean length of stay, days (SD)	15.7 (53.7)	75.7 (138)	25.7 (80.9)	10.7 (31.9)	50.0	2.95
Median length of stay, days (IQR)	8 (4–17)	51 (30–88)	16 (9–30)	6 (3–12)	-	-
In-hospital mortality, n (%)	334 383 (4.30)	21 459 (22.9)	136 495 (6.26)	176 429 (3.21)	(16.6)	3.66
All hospitalization costs, US$	49 448 074 206	3 148 043 318	21 358 213 253	24 941 817 635	-	-
All length of stay, days	121 635 824	7 105 175	55 984 570	58 546 079	-	-

SD, Standard Deviation; IQR, Interquartile Range; MRSA, Methicillin-resistant *Staphylococcus aureus*

^a^ Anti-MRSA drug group: Patients administered anti-MRSA drugs for four days or more

^b^ Control group: Patients administered antibiotics (excluding anti-MRSA drugs) for four days or more

^c^ Non-infection group: All other patients not included in the anti-MRSA drug group or the control group

^d^ Mean difference: Mean outcome value of the anti-MRSA drug group minus the mean outcome value of the control group

^e^ Mean ratio: Mean outcome value of the anti-MRSA drug group divided by the mean outcome value of the control group

### Outcomes in surgical and non-surgical inpatients

Table 2 shows the comparison of outcomes between surgical and non-surgical inpatients. In the surgery cohort, the median hospitalization costs and LOS in the anti-MRSA drug group were US$29 569 and 60 days, respectively, which were higher than those of the control group. In the non-surgery cohort, the mean hospitalization costs and LOS in the anti-MRSA drug group were US$24 007 and 63.2 days, respectively, which were also higher than those of the control group. The mean difference in hospitalization costs and LOS between the anti-MRSA drug group and the control group in the surgery cohort were US$28 588 and 58.9 days, respectively. In the surgery cohort, the mean ratios of the hospitalization costs and LOS in the anti-MRSA drug group to the control group were 3.26 and 3.10, respectively; in the non-surgery cohort, the corresponding ratios were 3.42 and 2.68, respectively.

**Table 2:** Demographic characteristics of the surgical and non-surgical inpatients.

	Surgical Inpatients^a^ n = 3 486 003				Non-surgical Inpatients n = 4 286 047			
	Anti-MRSA drug group^b^	Control group^c^	Mean Difference^d^	Mean ratio^e^	Anti-MRSA drug group^b^	Control group^c^	Mean Difference^d^	Mean ratio^e^
Number of hospitals, n	1085	1131	-	-	1104	1132	-	-
Number of patients, n (%)	49 787 (1.43)	1 028 484 (29.5)	-	-	44 051 (1.03)	1 153 343 (26.9)	-	-
Mean age, years (SD)	65.6 (20.9)	64.6 (20.2)	-	-	68.2 (21.0)	61.9 (28.4)	-	-
Female sex, n (%)	18 262 (36.7)	500 009 (47.5)	-	-	17 317 (39.3)	526 393 (45.6)	-	-
Mean hospitalization costs, US$ (SD)	41 989 (52 388)	13 401 (14 803)	28 588	3.26	24 007 (36 606)	7020 (9681)	16 987	3.42
Median hospitalization costs, US$ (IQR)	29 596 (16 753–49 789)	8771 (5246–15 581)	-	-	14 721 (8049–26 079)	4306 (2606–8154)	-	-
Mean length of stay, days (SD)	86.8 (149)	27.9 (82.2)	58.9	3.10	63.2 (125)	23.6 (79.7)	39.6	2.68
Median length of stay, days (IQR)	60 (36–100)	18 (10–34)	-	-	43 (24–74)	14 (8–27)	-	-
In-hospital mortality, n (%)	9471 (19.0)	33 888 (3.29)	(15.7)	5.77	11 988 (27.2)	102 607 (8.90)	(18.3)	3.06

SD, Standard Deviation; IQR, Interquartile Range; MRSA, Methicillin-resistant *Staphylococcus aureus*

^a^ Surgical inpatients: Patients who had undergone surgery during hospitalization

^b^ Anti-MRSA drug group: Patients administered anti-MRSA drugs for four days or more

^c^ Control group: Patients administered antibiotics (excluding anti-MRSA drugs) for four days or more

^d^ Mean difference: Mean outcome value of the anti-MRSA drug group minus the mean outcome value of the control group

^e^ Mean ratio: Mean outcome value of the anti-MRSA drug group divided by the mean outcome value of the control group

### Disease burden

Table 3 shows the total incremental burden of hospitalization costs, LOS, and mortality of the anti-MRSA drug group relative to the control group. We calculated the burden in two populations, where Population A comprised 7 772 050 inpatients from 1133 hospitals in this database and Population B comprised 9 203 194 inpatients in all hospitals (n = 1584) that have adopted the DPC/PCPS.

**Table 3:** Estimation of the incremental disease burden of MRSA infections.

Number of hospitals	Population A^a^	Population A (Adjusted) ^b^	Population B^c^
	n = 1133	n = 1133	n = 1584
All inpatients	n = 7 772 050	n = 7 772 050	n = 9 203 194
Total incremental hospitalization costs, US$	1 688 325 549	162 077 310	1 999 213 536
Total incremental length of stay, days	3 669 251	3 638 268	4 344 906
Total incremental deaths, n	12 112	12 124	14 342
Surgical inpatients^d^	n = 3 486 003	n = 3 486 003	n = 4 127 915
Total incremental hospitalization costs, US$	1 074 390 814	103 543 097	1 272 228 960
Total incremental length of stay, days	2 199 150	2 194 888	2 604 101
Total incremental deaths, n	5477	5450	6486
Non-surgical inpatients	n = 4 286 047	n = 4 286 047	n = 5 075 279
Total incremental hospitalization costs, US$	613 934 735	58 534 213	726 984 576
Total incremental length of stay, days	1 470 101	1 443 380	1 740 805
Total incremental deaths, n	6635	6674	7 857

^a^ Population A: All inpatients in the anti-MRSA drug group in the database used in this study

^b^ Population A (Adjusted): Population A adjusted for age (Three categories of ≤64 y, 65–74 y, and ≥75 y) and sex

^c^ Population B: All inpatients in the anti-MRSA drug group in all hospitals throughout Japan that adopted the DPC/PCPS. Burden estimations were extrapolated with reference to the number of patients from a public survey.

^d^ Surgical inpatients: patients who had undergone surgery during hospitalization

In Population A, the total burden of hospitalization costs, LOS, and mortality attributable to MRSA were US$1 688 325 549, 3 669 251 days, and 12 112 deaths, respectively. In Population B, the total burden of hospitalization costs, LOS, and mortality attributable to MRSA were US$1 999 213 536, 4 344 906 days and 14 342 deaths, respectively; this represented 3.41%, 3.02% and 3.62%, respectively, of the cumulative hospitalization costs, LOS, and mortality in Population B. The results of Population B were similar to the results of Population A that had been adjusted for age and sex.

### O/E ratios of estimated MRSA incidence

**Fig 1:**
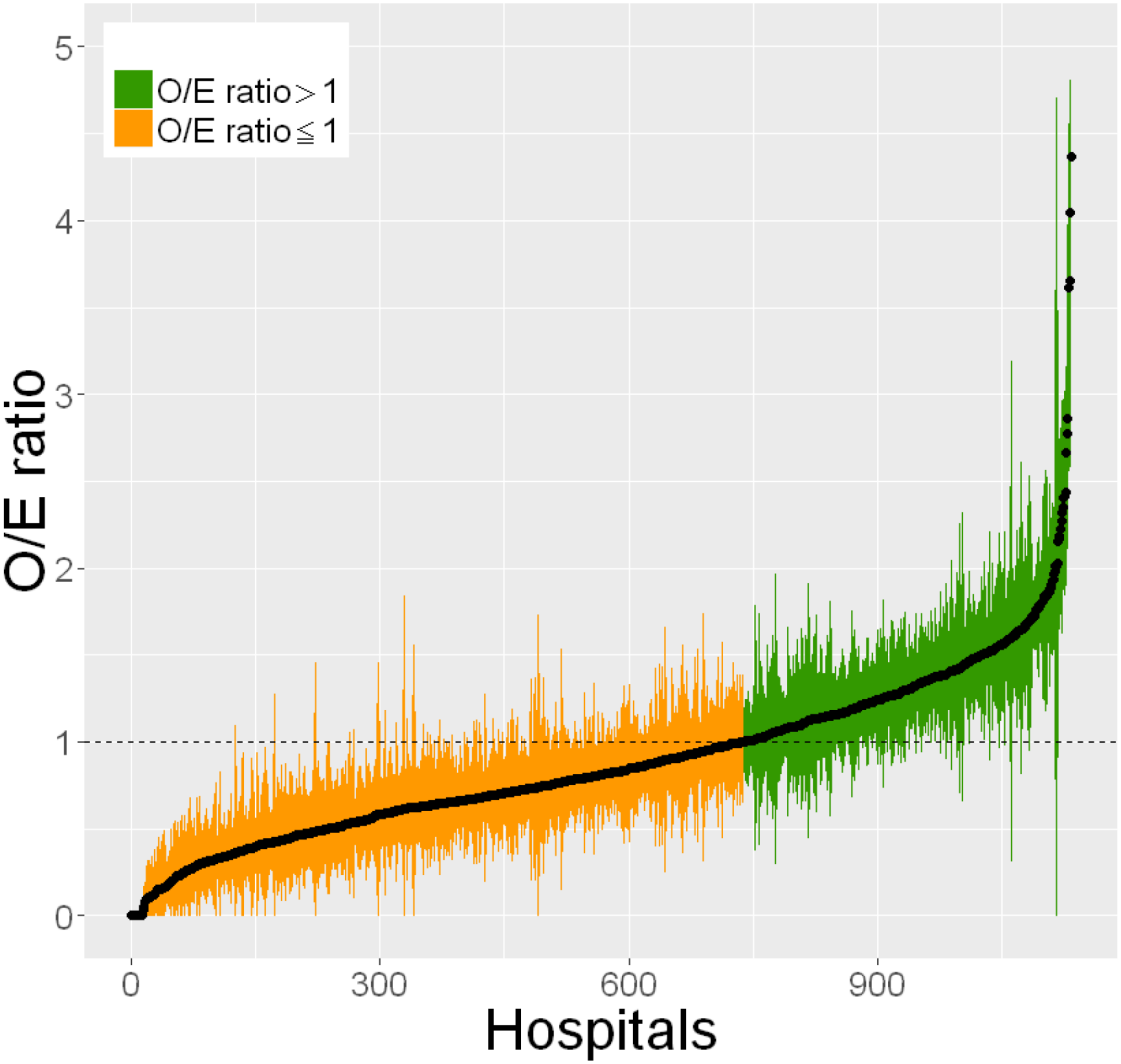
Hospital comparison of observed-to-expected ratios of estimated MRSA incidence. The horizontal axis represents individual hospitals and the vertical axis represents the observed-to-expected (O/E) ratios. The dots and bars indicate the O/E ratios and the 95% confidence intervals, respectively, of estimated MRSA incidence in each hospital.

Fig 1 shows the hospital-level O/E ratios of estimated MRSA incidence and the 95% confidence intervals (95%CI) in ascending order. Among the hospitals, the highest O/E ratio was 4.37 (95%CI: 3.35–5.44) and the lowest O/E ratio (14 hospitals) was zero; the latter indicated that the hospitals had not used anti-MRSA drugs for more than four days in any of their patients. There were 499 hospitals where the upper 95% CI of the O/E ratio was less than one, and 231 hospitals where the lower 95% CI of O/E ratio was greater than one. This indicated that the use of anti-MRSA drugs was less than expected in the former and more than expected in the latter. Overall, the results revealed large variations in O/E ratios among the hospitals.

## Discussion

In this nationwide study of hospitals in Japan, we estimated the incidence of MRSA infection to be approximately 1.21% of all inpatients in our database, and the O/E ratios of estimated MRSA incidence varied widely among hospitals. When compared with the control group, the mean ratios of the anti-MRSA drug group were 3.43 for hospitalization costs, 2.95 for LOS, and 3.66 for in-hospital mortality. The hospitalization costs in surgical patients with MRSA were approximately twice that of their non-surgical counterparts. The total incremental burden of MRSA in 9.20 million inpatients from all 1584 hospitals that have adopted the DPC/PDPS was estimated to be approximately US$2.00 billion (3.41% of all hospitalization costs), 4.34 million days (3.02% of all LOS), and 14.3 thousand deaths (3.62% of all in-hospital mortality).

A previous study investigated the economic burden of inpatients by calculating the difference in costs and LOS between patients with and without AMR infections: in an analysis of 1253 inpatients in the US, Roberts et al. reported that the mean medical costs and LOS were US$56 745 and 26.4 days in patients with AMR infections and US$13 210 and 8.0 days in patients without AMR, respectively [6]. The mean ratios of medical costs and LOS in patients with AMR infections to those without infections were 4.3 and 3.3 (standard error not available), respectively. These values were slightly higher than our results. We posit that the reason for this difference may be that the composition of AMR infection types in Japan is different from that of the US; in the US, vancomycin-resistant enterococci (VRE) accounts for 30.9% of all AMR infections, and is exceeded only by MRSA (43.1%). Because the burden of VRE and other AMR infections has a generally higher impact than MRSA infections [21], Japanese healthcare facilities (where the vast majority of AMR infections are due to MRSA) may be less susceptible to the effects of these infections. The substantial difference in LOS between the two studies may be due to a fundamental difference in average LOS duration between the two countries. While hospitalizations are generally shorter in the US [22], Japan has the longest mean LOS duration among the member countries of the Organisation for Economic Co-operation and Development [23].

Our analysis of the differences between surgical and non-surgical patients showed that surgical patients with MRSA infections had a greater impact on disease burden than non-surgical patients with MRSA infections. Roberts et al. reported that hospitalization costs and LOS in surgical patients were approximately twice that of medical patients [6], which was similar to our findings. A possible reason for this observation is that unit prices in surgical cases are generally higher than in non-surgical cases. Another possibility is that MRSA infections in surgical cases may require reoperation or result in deep infections (such as osteomyelitis), which induce a heavier clinical and economic burden [19, 24]. In addition, the burden of MRSA infections varied widely among the different diseases. For example, MRSA infections had the highest impact in non-surgical inpatients with myeloid leukemia (S1 Table), and also had a major impact on patients diagnosed as having “other sepsis” in both surgical and non-surgical cases. These high-burden diseases and surgery cases require further studies to explore the underlying factors that affect disease burden.

Our findings from the O/E ratios showed that estimated MRSA incidence varied widely among hospitals. Many of the existing studies regarding AMR infections were conducted using data from one or several hospitals. The Infectious Diseases Society of America reported that the burden of AMR infections in the US was $US21-34 billion based on the results of three studies [6–8, 25]. However, as those estimates of AMR infection burden were calculated using data from only one facility, the findings may be biased due to the inevitable variations in AMR infection incidence. As AMR infection incidence has been shown to vary among facilities as well as regions [1, 2, 26], nationwide surveillance system of AMR infections for the appropriate evaluation and prevention of these infections is needed. The unique method described here may have applications for the nationwide monitoring of MRSA infections due to the ease of collecting administrative claims data and the wide range of available information. We believe it will be relatively simple to quickly and inexpensively support the surveillance of MRSA infections using this nationwide database.

### Limitations

Although this study was strengthened by the large sample size across multiple facilities, our study has several limitations. First, the DPC database does not provide microscopy or laboratory-based information on the infecting pathogens. Our findings may include a degree of selection bias as MRSA cases were identified through the use of anti-MRSA drugs. For example, it is possible that anti-MRSA drugs were administered not only to treat MRSA, but also to treat other pathogens such as penicillin-resistant *S*. *pneumoniae*, as previously described [4]. In order to enable the identification of etiologic agents, we are exploring the use of microbiological data in a format developed by the Japan Nosocomial Infections Surveillance program [5] for future analyses. Second, we did not consider the differences in baseline characteristics, such as severity and comorbidities, between the anti-MRSA drug group and the control group when estimating the burden of MRSA infections. Because these characteristics may influence hospitalization costs, LOS, and mortality [15, 27], this may have introduced bias into the estimation of disease burden. Third, due to data limitations, the control group may include other multidrug-resistant bacteria such as penicillin-resistant *Streptococcus pneumoniae* and carbapenem-resistant *Enterobacteriaceae*. This may have resulted in an underestimation of the costs attributable to MRSA infections alone.

## Conclusions

In this study of inpatients in Japan, we quantified the clinical and economic burden caused by MRSA infections. Our findings indicate that these infections result in substantial clinical and economic burden through higher hospitalization costs, longer LOS, and higher in-hospital mortality rate. These estimates may provide a benchmark for subsequent studies and inform policymakers for the development and application of infection prevention measures.

## Supporting information

S1 Table Top 10 diseases with the highest incremental hospitalization costs due to MRSA infections in surgical and non-surgical inpatients. (DOCX)
