# Supplementary material for: Estimating the disease burden of methicillin-resistant Staphylococcus aureus in Japan: Retrospective database study of Japanese hospitals
Source: PLoS One. 2017 Jun 27;12(6):e0179767. doi: 10.1371/journal.pone.0179767 (PMC5487039; doi:10.1371/journal.pone.0179767)
Supplement: S1 Table — (DOCX) [file pone.0179767.s001.docx]

**S1 Table.** Top 10 diseases with the highest incremental hospitalization costs due to MRSA infections in surgical and non-surgical inpatients

| All Inpatients (n=7 772 050) | | | | | | | |
| --- | --- | --- | --- | --- | --- | --- | --- |
| Surgical Inpatients ^a^ (n=3 486 003) | | | | Non-surgical Inpatients (n=4 286 047) | | | |
| ICD10 code | Disease | MRSA cases,  n | Total incremental  hospitalization  costs, US$ | ICD10 code | Disease | MRSA cases,  n | Total  incremental  hospitalization  costs, US$ |
| A41 | Other sepsis | 3412 | 75 441 835 | C92 | Myeloid leukemia | 4180 | 82 420 907 |
| I71 | Aortic aneurysm and dissection | 1881 | 61 107 060 | C91 | Lymphoid leukemia | 1346 | 51 253 410 |
| P07 | Disorders of newborn related to short gestation and low birth weight,  not elsewhere classified | 504 | 25 849 818 | A41 | Other sepsis | 3957 | 43 996 725 |
| D65 | Disseminated intravascular coagulation | 1071 | 25 429 107 | D46 | Myelodysplastic syndromes | 1056 | 30 272 588 |
| I70 | Atherosclerosis | 1143 | 21 301 397 | J69 | Pneumonitis due to solids and liquids | 3422 | 26 358 899 |
| I21 | ST elevation and non-ST elevation myocardial infarction | 727 | 21 246 490 | C83 | Non-follicular lymphoma | 1336 | 23 930 328 |
| T81 | Complications of procedures, not elsewhere classified | 1341 | 21 110 482 | J15 | Bacterial pneumonia, not elsewhere classified | 2451 | 21 369 997 |
| N18 | Chronic kidney disease | 1030 | 19 159 299 | J18 | Pneumonia, unspecified organism | 1173 | 12 537 491 |
| I42 | Cardiomyopathy | 134 | 18 820 749 | I50 | Heart failure | 1112 | 12 380 944 |
| I50 | Heart failure | 625 | 18 781 211 | D65 | Disseminated intravascular coagulation | 1147 | 11 396 522 |

^a^ Surgical inpatients: patients who had undergone surgery during hospitalization
